# Long QT Syndrome: LQT3 Variant Presenting in a Bradycardic Newborn

**DOI:** 10.7759/cureus.76430

**Published:** 2024-12-26

**Authors:** Catarina Afonso, Débora A Mendes, Rita V Queirós, Catarina M Serra, Sérgio Laranjo

**Affiliations:** 1 Family Medicine, USF (Unidade de Saúde Familiar) Afonsoeiro, Unidade Local de Saúde do Arco Ribeirinho, Montijo, PRT; 2 Pediatrics, Unidade Local de Saúde do Médio Tejo, Torres Novas, PRT; 3 Pediatrics, Centro Hospitalar Barreiro Montijo, Unidade Local de Saúde do Arco Ribeirinho, Barreiro, PRT; 4 Pediatric Cardiology, Centro Hospitalar Lisboa Central, Unidade Local de Saúde de São José, Lisbon, PRT

**Keywords:** bradycardia, long qt syndrome, lqt3 variant, newborn, propranolol

## Abstract

Long QT Syndrome (LQTS) is a rare hereditary canalopathy, characterized by prolonged ventricular repolarization, which can lead to malignant tachyarrhythmias at a young age. Treatment typically involves healthy lifestyle changes and β-blocker therapy. In specific cases, the implantation of an implantable cardioverter defibrillator (ICD) can be an option. In this report, we present the case of a newborn presenting with bradycardia in his initial screening examination, who was subsequently diagnosed with LQTS, specifically variant LQT3, a rare finding.

## Introduction

Long QT syndrome (LQTS) is a rare hereditary arrhythmic condition caused by mutations in various genes that encode ion channels or auxiliary proteins [1-11]. These mutations can result in either a gain or loss of function, interfering in myocardial repolarization. This disruption causes a prolongation of the action potential, evident as a long QT interval on an electrocardiogram (EKG), which can lead to syncope, cardiac arrest, or even death. 

There are two known hereditary forms of LQTS: a rare autosomal recessive variant associated with congenital deafness, called Jervell Lange-Nielsen syndrome, and, the most common, an autosomal dominant without congenital deafness, known as Romano-Ward syndrome [1-4].

Currently, LQTS is associated with 17 different genes [2,5]. Each mutation leads to specific clinical manifestations, different triggers for cardiac events, and consequently distinct treatment responses [2]. These genotype-phenotype relationships have been described for the three most common subtypes: LQTS type one (LQT1), type two (LQT2), and type three (LQT3), which together account for approximately 75% of all LQTS patients [3,5]. Notably, around 20% of all patients with a clinical diagnosis do not have identifiable mutations [3], and about 50% of those with a mutation are asymptomatic [6]. Among patients with a confirmed genotype, about 85% inherited the mutation from a parent, while the remaining 15% have a de novo mutation [8].

Mutations in the *KCNQ1* gene, which encodes the alpha subunit (KvLQT1) of the potassium channel, are found in the LQT1 variant, the most prevalent form [5,7]. The *KCNH2* gene encodes the alpha subunit of the hERG (human Ether-à-go-go Related Gene) potassium channel in the LQT2 variant [5,7]. In LQT3, mutations in the *SCN5A* gene affect a cardiac sodium channel protein [4,7]. Studies revealed that these mutations lead to an increased late sodium current, resulting in a gain of function that prolongs the action potential duration [7].

Regarding triggers for cardiac events, these differ by LQTS subtype [3,7,8]. In LQT1, adrenergic stimuli like physical activity (e.g., swimming and diving) can trigger events [3,7,8]. LQT2 is associated with emotional stress and sudden exposure to auditive noises (e.g., telephone rings, alarm clocks, and crying babies) [3]. For LQT3, about 65% of events occur during sleep or rest, often without emotional triggers, with bradycardia being a common precipitant [3,6].

Although many individuals remain asymptomatic throughout their lives, when symptoms do occur, they can vary significantly, with genotype playing a role in the severity of the manifestations. Symptoms may include syncope, seizures, bradycardia, cardiorespiratory arrest, and sudden death [3], typically presenting between the ages of 12 and 18 years [3]. Early diagnosis can be challenging, as QT prolongation in newborns can be transient and not indicative of an LQTS [1,3,12].

Polymorphic ventricular tachycardia is the entity responsible for the syncope, and these episodes can be accompanied by tonic-clonic movements therefore misdiagnosed as epilepsy [3]. Some patients may also present with auriculoventricular (AV) block, atrial arrhythmia, and bradycardia [3].

Initial diagnostic evaluation includes a complete clinical history with familiar data, physical examination, 12 lead EKG evaluation with corrected QT (QTc) interval calculation, effort EKG, 24-hour Holter, and genetic testing [2,3,5]. The diagnosis can also be made using the Schwartz score, which includes the measurement of the QTc interval and other clinical and familial factors [2,3,6,8]. QTc measure is obtained by using the Bazett formula, dividing the QT interval by the square root of the RR interval, $QTc =\frac{QT}{\sqrt{RR}}$, ideally from V5 or DII derivations [4]. QTc normal interval is ≤440 ms, so > 440 ms is considered prolonged [2]. Patients with a Schwartz score higher than 3.5, in the absence of a hereditary cause for QT prolongation, have a high probability of a positive LQTS genetic test [3,6]. For those with scores between 1 and 3, serial EKGs, 24-hour Holter monitoring, and genetic testing are recommended to identify subclinical forms [3,6]. If the score is less than 1, individuals are generally considered to be without the disease [2,3,6].

Genetic testing is useful when diagnosis is uncertain and also for assessing a prognosis and therapeutic response [3]. Since treatment approaches can vary, options available include lifestyle modifications, pharmacologic treatment focusing on β-blockers, implantable cardioverter defibrillators (ICDs), and surgical intervention such as left cardiac sympathetic denervation (LCSD) [3].

Additionally, it appears possible to stratify the risk and predict LQTS genotype based on prenatal rhythms [13]. It is known that sinus bradycardia, defined as a heart rate percentile < 3 for gestational age, is the most common fetal arrhythmia associated with LQTS. Torsades de pointes and/or second-degree AV block can also occur as fetal arrhythmia characteristic of this syndrome [13]. LQTS intrauterine and neonatal manifestations are associated with a higher cardiac risk, particularly when there is an AV block and a significant QT prolongation exceeding 600 ms [14].

Currently, β-blockers (such as propranolol at 2-4 mg/kg/day) are the first-line treatment, showing a reduction of the arrhythmic events from 0.97 to 0.31 events per patient per year [6]. Among the three main subtypes of LQTS, β-blockers are particularly effective in LQT1, where adrenergic stimuli trigger the arrhythmia [6]. Preliminary studies have also shown a protective effect from β-blockers in LQTS carriers [6]. Symptoms onset in the first year of life or a history of cardiac arrest constitute a higher risk, potentially implicating the necessity of an ICD, associated with left cardiac sympathectomy adjuvant to β-blocker treatment [2,3,6].

## Case presentation

We present the case of a full-term male newborn, resulting from a well-supervised pregnancy, with a family history that includes a two-year-old consanguineous sister diagnosed with trisomy 18, an eight-year-old maternal sibling with asthma, and a 12-year-old maternal sibling with a history of cleft palate. The mother had a prior history of treated hepatitis C, with a viral load of 13.56 in the third trimester, with positive antibodies for hepatitis C virus (HCV) but negative for the antigen. Additionally, she reported smoking seven to eight cigarettes daily and had a previous history of drug dependence. The newborn, appropriate for gestational age, was delivered by a non-instrumental vaginal birth. Following an initial Apgar score of 4, resuscitation was performed, leading to improved scores of 9 and 10 at the five- and ten-minute marks, respectively.

In the first screening examination, at approximately 11 hours of life, the newborn presented bilateral epicanthic folds, ankyloglossia, and bradycardia, with wide and symmetrical femoral pulses, without edema, jugular distension, or other changes. On neurologic examination, he was less reactive but with normal reflexes and normal postures. Vital signs were assessed, showing a heart rate (HR) of 60 to 74 beats per minute (bpm), which rose up to 100 bpm when crying, peripheral oxygen saturation of 98%, and blood glucose of 60mg/dL. Blood pressure was normal in all four limbs and without significant differences.

The newborn did an analytical evaluation that revealed no changes and an EKG that showed sinus bradycardia, with a prolonged QT interval, measured at 0.533 seconds using Bazzet's formula.

**Figure 1:**
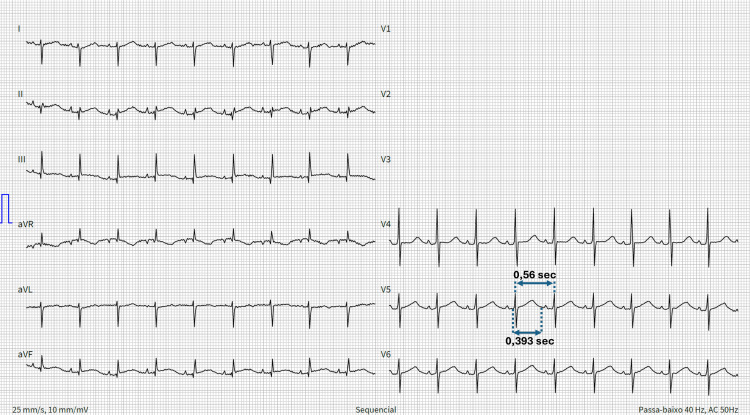
Patient's EKG showing a prolonged QT interval with QT 393 ms (the shorter two-headed arrow), RR 560 ms (the longer two-headed arrow), resulting in QTcB 524 ms.

The patient was referred to the Pediatric Cardiology Center where a transthoracic echocardiogram was performed and revealed to be normal. EKG was repeated (Figure 1) and confirmed the diagnosis of LQTS with sinus rhythm, HR 100 bpm, QRS axis in the right lower quadrant, normal PR interval, narrow QRS, and prolonged QT interval with QTcB 524 ms. A urine and blood toxicology screening were performed, both negative, and blood analysis was repeated, with normal thyroid-stimulating hormone (TSH) and no other alterations, except a positive HCV antibody.

Upon diagnosis, therapy with propranolol was initiated and optimized up to 1.6mg/kg/day. A Holter monitoring was performed with permanent sinus rhythm, mean HR 112 bpm and QTc 450 ms. The patient was discharged to the home hospital with an EKG that showed HR 98 bpm, QRS axis in the right lower quadrant, narrow QRS, and prolonged QT interval with QTc 405-455 ms.

During the rest of the hospitalization, he had a favorable weight gain, occasionally experiencing bradycardias of HR 80 bpm during breastfeeding with rapid spontaneous recovery. The newborn passed all other recommended screenings, including hearing screening, and red eye reflex testing, and was vaccinated against hepatitis B virus (HBV) before discharge.

At 22 days of age, he was evaluated in a pediatric arrhythmology appointment. The echocardiogram was normal and the EKG was in sinus rhythm, FC 149 bpm, and QTc 440-455ms. It was decided to maintain the medication with propranolol 1.5 mg three times a day (~ 1.4mg/kg/day).

The patient was reevaluated at two months of age, with a good weight (~ 5kg), and medicated with about 1 mg/kg/day of propranolol. The EKG revealed QTc 415-440 ms and Holter showed a permanent sinus rhythm and adequate chronotropic variation, two monomorphic and isolated ventricular ventricular extrasystoles, QTc 441 ms, without other changes. The dosage was not changed and the suspension of propranolol was considered at the next appointment. A genetic study was performed.

At four months of age, the genetic study revealed a variant in hererozigotia of the *SCN5A* gene (indeterminate meaning) providing for a functional impact (by predictive computational methods), so the dose of propranolol was adjusted from ~ 0.6 mg/kg/day (for the current weight of 6760 g) to approximately 1.5 mg/kg/day (3 mg three times a day).

The patient maintained the follow-up with propranolol dose adjustments to approximately 1.7mg/kg/day.

At the 10-month follow-up, he remained asymptomatic, Holter showed permanent sinus rhythm and adequate chronotropic variation, two double supraventricular extrasystoles, QTc 420 ms, without other changes. A propranolol dose adjustment was made to ~ 2.5mg/kg/day (7.5 mg three times a day). The patient will maintain follow-up in pediatric arrhythmology appointments with serial Holters.

## Discussion

Clinical presentation of LQTS can range from asymptomatic individuals to those experiencing recurrent syncope or even sudden death [1-11]. EKG may reveal extrasystoles or ventricular tachycardia [2,4]. There are known more than 500 mutations distributed by diverse genes involved in LQTS [4]. For the diagnosis, the Schwartz score uses the familiar history, EKG alterations, and pathological background as syncopes [1-11].

**Figure 2:**
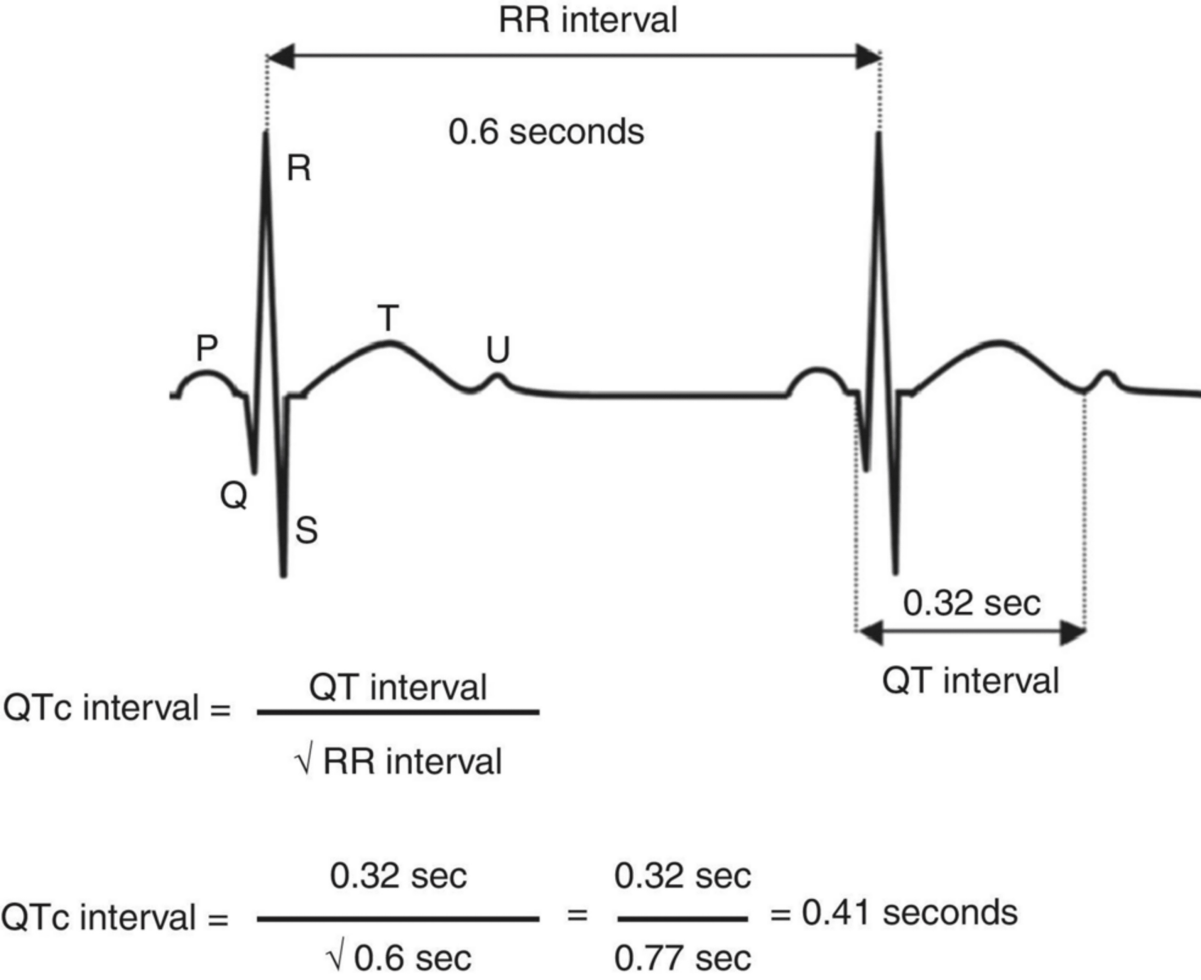
Example of the calculation of corrected QT interval by Bazzet's formula Image Source: Rodríguez-Balderrama et al., 2015 [4]; Under Creative Commons licence, Attribution-Non-Commercial-NoDerivatives 4.0 International, CC BY-NC-ND 4.0

In this case, we present an asymptomatic patient, born after a supervised pregnancy with no reported fetal cardiac rhythm issues, and bradycardia noted only upon auscultation as a potential concern. The patient presented a Schwartz score of 3.5 at diagnosis, based solely on QTc 524 ms and low HR for age (60-74 bpm) (Table 1), and secondary causes for QT prolongation were not identified. The QTc was calculated by the Bazett formula as shown in Figure 2. Therefore serial EKGs and Holter monitoring were necessary, along with genetic testing, which revealed a variant in heterozygosity of the *SCN5A* gene, normally associated with the LQT3 variant (Table 2) but, in this case, with unknown meaning, although predictive computational methods predict this variant has a functional impact [4,7].

**Table 1:** Schwartz score (actualized at 2011): diagnostic criteria LQTS In Schwartz score, QTc is calculated by the Bazett formula where QTc=QT/√RR. Bradycardia for a particular age means rest heart rate (HR) lower than percentile 2 for the same age. Table Source: Kun and Ping, 2021 [2]; Under Creative Commons licence, CC-BY-NC-ND

ECG findings	Points
QTc	
> 480 ms	3
460-470 ms	2
450 (male) ms	1
4 min recovery QTc after exercise test ≥ 480 ms	1
Torsades de pointes	2
T-wave alternans	1
Notched T wave in 3 leads	1
Low heart rate for age	0.5
Clinical history	
Syncope	
With stress	2
Without stress	1
Congenital deafness	0.5
Family history	
A. Family members with definite LQTS	1
B. Unexplained sudden cardiac death	0.5

**Table 2:** Congenital LQTS genes and affected ionic current Table Source (Original in Spanish): Fragata et al., 2016 [6]; Open access LQTS: long QT Syndrome

LQTS type	Gene		Protein		Current			Frequency (%)
Romano-Ward								
LQT1	KCNQ1		Kv7.1		↓|Ks			40-55
LQT2	KCNH2		Kv11.1		↓|Kr			30-45
LQT3	SCN5A		Nav1.5		↑|Na			5-10
LQT4	ANKB		Ankyrin		↓ Coordination of Ncx, Na/K ATPase			Rare
LQT5	KCNE1		MinK		↓|Ks			
LQT6	KCNE2		MiRP1		↓|Kr			
LQT7	KCNJ2		Kir2.1		↓|K1			
LQT8	CACNA1C		Cav1.2		↑|Ca			
LQT9	CAV3		Caveolin 3		↑|Na			
LQT10	SCN4B		Sodium channel β4-subunit		↑|Na			Very rare
LQT11	AKAP9		Yotiao		↓|Ks			
LQT12	SNTA1		Syntrophin-α1		↑|Na			
LQT13	KCNJ5		Kir3.4		↓|K-Ach			
LQT14	CALM1		Calmodulin 1		Dysfuntional Ca^2+^ signaling			Rare
LQT15	CALM2		Calmodulin 2					
Jervell and Lange-Nielsen								
JLN1		*KCNQ1*		Kv7.1		↓|Ks	Rare	
JLN2		*KCNE1*		MinK				

Currently, patients diagnosed with LQTS, regardless of symptomatology, are initiated on treatment with β-blockers, besides being advised to avoid intense physical activities and medications that prolong cardiac repolarization (Table 3) [4].

**Table 3:** List of medication which prolong cardiac repolarization and, consequently, the QT interval. Table Source: Rodríguez-Balderrama et al., 2015 [4]; Under Creative Commons license, Attribution-Non-Commercial-No Derivatives 4.0 International, CC BY-NC-ND 4.0

List of medications which prolong QT interval
Amantadine
Azithromycin/erytromycin
Chloral hydrate
Ciprofloxacin
Cisapride
Domperidone
Furosemide
Metronidazole
Trimepthoprim/Sulphamethoxazole

In those patients with high sudden death risk, namely AV blocks, ventricular tachycardia in torsade de pointes, and heart arrest survivors, an ICD should be implanted [2-6]. Every patient diagnosed with LQTS should be accompanied by a multidisciplinary team, including cardiologists and geneticists [4]. Genetic testing is crucial as it can impact the treatment according to the mutations found [4]. β-blocker therapy, such as propranolol (2-4 mg/kg/day) and nadolol (1-1.5 mg/kg/day) are used in the three main LQTS genotypes but they are more effective in LQT1 due to its pathogenesis, adrenergic stimulation [1]. β-blockers are less effective in LQT2 than in LQT1, possibly due to the reduction of adrenoreceptors α1A IKr mediated [1]. Studies have revealed that β-blockers are also protectors on LQT3 patients [1]. In the present case, the patient was diagnosed with an LQT3 variant and showed disease control with propranolol in a weight-adjusted dose.

As adjuvant therapy, some medications are used in specific genotypes [1]. In LQT2, potassium oral supplements can have a meaningful role, especially during acute periods of diarrhea, and mexiletine can be used in LQT3 [1]. Recently ranolazine, an antianginal medication, has been an option for the treatment of patients with LQTS because it blocks the late sodium current [1]. Until now there is only one clinical study, which includes a small number of patients, on the short-term effectiveness of ranolazine in LQT3 [1].

In the current case, the patient remains medicated with propranolol (currently with a dose of 2.5 mg/kg/day), is clinically stable, and has regular follow-ups in pediatric arrhythmology appointments with serial Holter monitoring.

## Conclusions

This case underscores the importance of thorough clinical examination of newborns, both during their time in maternity care as well as in subsequent health visits, with strong focus on cardiac assessment and cardiac auscultation skills. Developing the ability to recognize normal HR in newborns allows early diagnosis and prompt treatment of arrythmias that could lead to sudden death. Furthermore the importance of the genetic testing in a LQTS diagnosis cannot be overstated as it enhances therapeutic decision-making and prognosis accuracy.
